# Bioactive Compounds and Traditional Uses of *Tripleurospermum disciforme* (C.A.Mey.) Sch.Bip.: A Comprehensive Study on Its Therapeutic Potential

**DOI:** 10.3390/molecules30183685

**Published:** 2025-09-10

**Authors:** Parvaneh Sheydaei, Susana Ferreira, Micaela Almeida, Alexandra Coimbra, Fatemeh Yousefbeyk, Eugenia Gallardo, Luiza Breitenfeld, Maria Emília Amaral, Ana Paula Duarte

**Affiliations:** 1 RISE-Health, Department of Medical Sciences, Faculty of Health Sciences, University of Beira Interior, Av. In-fante D. Henrique, 6200-506 Covilhã, Portugal; susana.ferreira@fcsaude.ubi.pt (S.F.); malmeida@fcsaude.ubi.pt (M.A.); alexandra.coimbra@ubi.pt (A.C.); egallardo@fcsaude.ubi.pt (E.G.); luiza@fcsaude.ubi.pt (L.B.); 2 Clinical Academic Centre of Beiras (CACB), Edifício UBImedical, Estrada Municipal 506, 6200-284 Covilhã, Portugal; 3 Department of Pharmacognosy, School of Pharmacy, Guilan University of Medical Sciences, Rasht 41346-14335, Iran; yousefbeyk@gums.ac.ir; 4 Laboratório de Fármaco-Toxicologia, UBIMedical, Universidade da Beira Interior, Estrada Municipal 506, 6200-284 Covilhã, Portugal; 5 Fiber Materials and Environmental Technologies Research Unit (FibEnTech-UBI), University of Beira Interior, Rua Marquês D’Ávila e Bolama, 6201-001 Covilhã, Portugal; mecca@ubi.pt

**Keywords:** ethnobotany, *Tripleurospermum disciforme*, UHPLC–timsTOF–MS, antimicrobial activity, antioxidant activity, MTT/Protein Assay

## Abstract

*Tripleurospermum disciforme* (C.A.Mey.) Sch. Bip. is known as the “Plain Chamomile” of the Asteraceae family, the most prominent plant family and one that has been studied extensively due to its numerous genera and species. In traditional herbal medicine, *T. disciforme* has been used to treat digestive, neurological, and skin disorders. This study aimed to document ethnobotanical knowledge and assess the pharmacological potential of medicinal plants, specifically *T. disciforme*, across the provinces of Guilan, Alborz, and Qazvin in Iran, through ethnobotanical surveys. These surveys identified the most utilized plant families as Lamiaceae, Asteraceae, and Theaceae, with *T. disciforme* cited by 42% of participants, emphasizing its significance in local traditional medicine. Given its high relative frequency of citation and reported medicinal applications, *T. disciforme* extracts were subjected to UHPLC–timsTOF–MS analysis for further phytochemical profiling and a series of biological assays. Several phenolic compounds such as neochlorogenic acid, caffeic acid, and p-hydroxyphenylethanol acetate were recurrently detected across extracts. The ethyl acetate extract demonstrated potent antioxidant activity in the DPPH assay (IC_50_ = 12.496 µg/mL) and exhibited antimicrobial activity against *Bacillus cereus* (MIC = 312 µg/mL). Additionally, the hexane extract revealed notable cytotoxic effects against MCF-7 human breast cancer cells. To the best of our knowledge, to date, this is the first investigation of *T. disciforme* to integrate ethnobotanical and ethnopharmacological approaches to medicinal plant research in these regions of Iran.

## 1. Introduction

Iran, with its expansive geography and diverse climate zones, is recognized as one of the most botanically rich countries in the world. Hosting more than 8000 identified plant species, including 1727 endemics, Iran ranks among the top ten countries globally in terms of floristic diversity [1,2]. This exceptional plant wealth provides fertile ground for the study of medicinal flora, fostering the intersection of traditional herbal wisdom and modern scientific exploration. One notable example is *Tripleurospermum disciforme* (C.A.Mey.) Sch.Bip., also known as plain chamomile, which belongs to the Anthemideae tribe within the Asteraceae family, one of the largest plant families globally, locally known as “babuneh Dashti”. This herbaceous species, which may be perennial or biennial, typically reaches heights of 10 to 70 cm [3]. It is taxonomically recognized by the homotypic synonyms *Chrysanthemum disciforme* C.A.Mey., *Chamaemelum disciforme* (C.A.Mey.) Vis., and *Matricaria disciformis* (C.A.Mey.) DC. [4]. Ancient Persian medico-pharmaceutical manuscripts reference various botanical names for “babuneh”, such as *Anthemis nobilis* L., *Anthemis wiedemanniana* Fisch. & Mey., *Matricaria chamomilla* L., and *Tripleurospermum disciforme* (C.A.Mey.) Schultz Bip. [5]. Today, the chamomile-like plant sold in Iranian herbal markets under the name “babuneh” is most commonly *T. disciforme*, which exhibits botanical features distinct from the more internationally known *Matricaria chamomilla* [6].

The genus *Tripleurospermum* includes around 40 species, primarily distributed across temperate regions of Europe and Asia, with additional representation in North Africa and North America [7,8,9]. Turkey is considered the primary biodiversity centre for this genus [9,10], while Russia harbours seventeen species [11], and six species are reported from Iran [12]. Ethnobotanical studies of *Tripleurospermum* species have demonstrated that they possess anti-inflammatory properties, alleviate gastric pain, serve as antiseptics, benefit hair care, and enhance memory [13]. Phytochemical studies of *Tripleurospermum* species have revealed a wide range of bioactive compounds, including terpenoids, alkanes, steroids, organic acids, and aromatic constituents [13,14].

In addition, triterpenoids such as taraxasteol, lupeol, and betulinic acid have been identified in *T. disciforme* (C.A.Mey.) Sch.Bip., with lupeol and betulinic acid reported to inhibit tubulin polymerization in gastric cancer cells (AGS) [15]. These compounds have also shown notable cytotoxic activity against diverse cancer cell lines, including breast, cervix, colon, prostate, lung, ovary, skin, hematopoietic, and lymphoid cancers [16,17,18], further highlighting the therapeutic relevance of *T. disciforme* (C.A.Mey.) Sch.Bip.

The therapeutic efficacy of *T. disciforme* is primarily attributed to its rich phenolic profile, which imparts anti-ulcer [19], antibacterial [20,21], antioxidant [22], antispasmodic, and antiseptic activities [23]. Despite its widespread presence across Iran, the rapid pace of technological and societal transformation poses a serious threat to the preservation of traditional herbal knowledge. As such, comprehensive documentation is imperative to safeguarding this cultural and pharmacological legacy [24].

Ethnobotany serves as a vital bridge between ancestral knowledge and contemporary science, focusing on the documentation, analysis, and interpretation of plant use within specific cultural and ecological contexts [8]. This study represents the most extensive investigation of *T. disciforme* to date and constitutes the first ethnobotanical and ethnopharmacological assessment of medicinal plants in the provinces of Guilan, Qazvin, and Karaj. By integrating indigenous knowledge with modern quantitative ethnobotanical methodologies, this research pioneers a systematic evaluation of regional medicinal flora through a set of analytical indicators, offering a foundational contribution to both the local and global herbal medicine literature. Additionally, the aerial parts of *T. disciforme* (C.A.Mey.) Sch. Bip were extracted using various solvents to isolate secondary metabolites. The resulting extracts were subsequently analyzed by ultra-high-performance liquid chromatography coupled with trapped ion mobility spectrometry and tandem mass spectrometry (UHPLC–timsTOF–MS) to assess their antioxidant, antimicrobial, and cytotoxic activities.

In this context, we hypothesize that *Tripleurospermum disciforme*, traditionally used as a medicinal plant, may contain bioactive metabolites with antioxidant, antimicrobial, and cytotoxic potential. To address this, ethnobotanical documentation was combined with phytochemical and biological analyses of specimens collected from Rasht, aiming to scientifically validate traditional knowledge and provide a basis for future pharmacological studies.

## 2. Results and Discussion

### 2.1. Ethnobotanical Surveys

In this ethnobotanical study, a total of 100 residents from the provinces of Guilan, Qazvin, and Alborz were interviewed. Participants were recruited through a convenience sampling strategy, based on voluntary participation at local herbal markets. The results indicated that individuals aged between 30 and 49 years represented the primary users of medicinal plants, accounting for 58% of respondents, whereas those aged over 70 showed the lowest rate of use, comprising only 1%. These findings are in line with ethnobotanical research conducted in Angola, where individuals within the same age range were identified as the most frequent users of medicinal plants [25].

Furthermore, this study revealed that 84% of medicinal plant users were natives of the Guilan, Karaj, and Qazvin provinces. The data also support previous findings indicating that women were the predominant consumers of medicinal plants compared to men [25]. Women constituted 65% of users, a trend that reflects their traditional roles in the preparation and administration of herbal remedies, knowledge that is often transmitted through generations, predominantly from mother to daughter. These results are consistent with ethnobotanical studies conducted in countries such as Algeria [26], Morocco [27], Angola [25], and Turkey [28], which have similarly reported that women possess greater awareness and knowledge regarding the traditional use of medicinal plants for treating various ailments.

**Figure 1:**
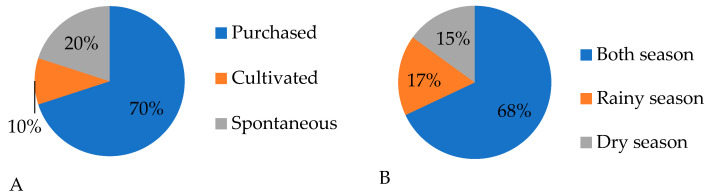
Use of medicinal plants according to forms of obtention (**A**) and season of collection (**B**).

Ethnobotanical studies reveal that a significant portion of people acquire medicinal plants by purchasing them at markets or specialized stores (Figure 1A). This trend is driven by several factors, including convenience, the elimination of the need for wild plant identification and collection, and the assurance of quality and authenticity in commercially available products. Moreover, the decline in traditional knowledge and limited expertise in identifying medicinal plants has led many individuals to favour pre-prepared forms of these plants over collecting raw plant materials, reflecting a cultural transition towards processed and commercialized products.

Ethnobotanical research further indicates that the use of medicinal plants reaches its peak during transitional seasons, particularly in both the rainy season and the dry season (Figure 1B). Our findings align with ethnobotanical studies from Angola, which also reported the highest usage of medicinal plants during these seasons [25].

**Figure 2:**
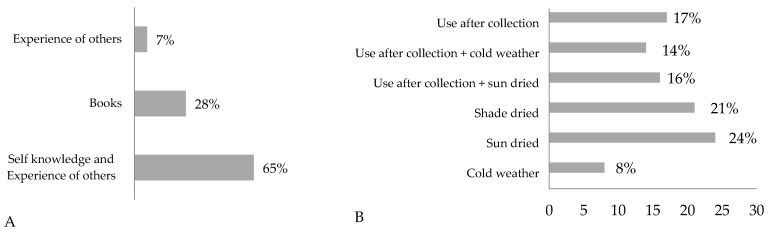
Use of medicinal plants according to information source (**A**) and plant conservation mode (**B**).

In the provinces of Guilan, Qazvin, and Karaj, the reliance on medicinal plants was predominantly influenced by communal knowledge and shared experiences, with 65% of users indicating that their use was based on the experiences of others (Figure 2A). A similar pattern was observed in Angola, where the traditional knowledge of medicinal plants was largely verified and transmitted through collective experience [25].

As for plant conservation practices, 21% of participants reported using shade-drying methods to preserve medicinal plants (Figure 2B), reflecting a common and effective approach in maintaining the quality and efficacy of herbal remedies. Sun-drying was another common practice, employed by 24% of individuals, as it facilitates medium- to long-term preservation. Meanwhile, 17% of the respondents used medicinal plants immediately after their collection, foregoing any preservation process.

**Figure 3:**
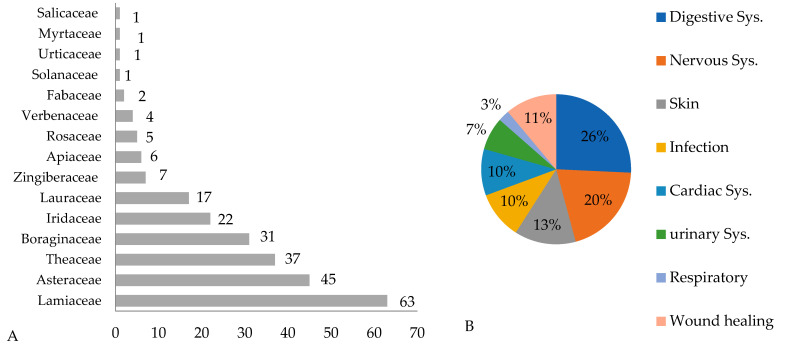
Most cited taxa (**A**) and therapeutic indication (**B**).

The present survey demonstrated that the most frequently cited plants belong to the Lamiaceae family (Figure 3A), which accounted for 63 mentions. This was followed by plants from the Asteraceae (45) and Theaceae (37) families. The Lamiaceae family is one of the most significant plant families in Iran, comprising 46 genera and 406 species, of which 165 are endemic. This family is known for its wide range of biological and medicinal applications [29,30].

According to our findings, the two families Lamiaceae and Asteraceae represent the most widely used medicinal plants. Similar results have been reported in other regions of Iran, including the forests of Arasbaran [31], where these families are dominant, in Sirjan, Kerman Province, where Lamiaceae is identified as the largest medicinal plant family [32], and in the Saravan region of Baluchistan, where Lamiaceae also plays a prominent role in traditional medicine [33]. Additionally, in Bojnourd, North Khorasan, Lamiaceae and Asteraceae were reported as the most commonly used medicinal plant families [34].

Based on the therapeutic indications of the medicinal plants, most were employed to treat disorders of the digestive system (26%), followed by those related to nervous system disorders (20%) (see Figure 3B). Additionally, the surveys frequently mentioned ailments associated with the skin, infections, and issues related to the cardiovascular, urinary, and respiratory systems, and for wound healing.

Moreover, a separate study conducted by [35] revealed that in Guilan Province, particularly in the regions of Amlash and Rudsar, the most commonly utilized medicinal plants belonged to the Lamiaceae family. These plants were primarily used to treat digestive disorders, accounting for 20% of reported cases. This trend aligns with findings from ethnobotanical investigations in other parts of Iran, such as Kohgiluyeh and Boyer-Ahmad Province [36], where the Asteraceae and Lamiaceae families are predominant, and digestive ailments represented the most frequently addressed health concerns. Similarly, research conducted by [37], in the village of Juban, also located in Guilan Province, confirmed that the main reason for medicinal plant use among locals was the treatment of digestive disorders. This pattern is not unique to Iran. In Turkey as well, the majority of medicinal plant applications were reported for gastrointestinal disorders [38,39].

Regarding preparation methods, infusion was the most preferred technique among users, employed in 45% of cases, followed by decoction (19%), use in natural form (15%), and maceration (13%). Comparable trends have been observed in other regions of Iran, where infusion and decoction are also the predominant methods of preparation [40].

These methods are often preferred due to their simplicity and the use of water as a solvent, as highlighted in previous studies [26,41]. Their ease of preparation makes infusion and decoction particularly popular techniques for extracting active compounds from medicinal plants. As for the parts of medicinal plants most frequently used, leaves alone (28%) and mixtures of leaves and flowers (17%) were the most commonly cited.

Furthermore, the highest proportion of medicinal plant use has been attributed to leaves, accounting for 39% in the Arasbaran forests of northern Iran [31], the Zangelanlo district in Northeast Iran [42], and Lorestan Province [43], and 31% in the Saravan region of Baluchistan, Iran [33]. In addition, the most common methods of preparation reported by local communities were infusions and decoctions, while poultices were the least commonly used. These patterns are consistent with our findings [31]. Similarly, ethnobotanical studies conducted in North Khorasan have also identified infusions and decoctions as the primary methods of medicinal plant use, in agreement with our results [34].

It is important to note that, although this approach provided direct access to key informants and facilitated the collection of relevant data, the sample size (n = 100) was relatively limited and no stratification by age, gender, or specific communities was performed. Consequently, the results should be interpreted with caution, as the data may not fully represent the wider population and may be subject to selection bias, particularly favouring individuals more actively involved in local herbal trade. Nevertheless, despite these limitations, the survey offers valuable exploratory evidence on regional practices and provides a foundation for future studies employing stratified or larger-scale sampling strategies.

#### Quantitative Statistical Analysis of Ethnobotanical Results

The Informant Consensus Factor (ICF) values ranged from 0 to 1, indicating the level of agreement among informants on plant use for specific ailments. Low values suggest diverse or uncertain knowledge, while high values reflect strong consensus and likely indicate culturally important and trusted remedies. The recorded traditional uses were grouped by therapeutic indications (Table 1).

The highest ICF value (0.85) was verified for the digestive system, followed by the nervous system (0.82) and wound healing (0.77). Although all ICF values were greater than 0.5, the lowest ICF was obtained for the urinary system.

The strong preference of the local population for specific medicinal plants, based on their therapeutic applications, is supported by high fidelity level (FL) values (Table 2).

For instance, *Mentha* spp. exhibited the highest FL value (100%), indicating its exclusive use for treating digestive ailments. This underscores a significant cultural reliance on *Mentha* spp. for gastrointestinal disorders. Traditionally, *Mentha piperita* has been employed to address biliary conditions, indigestion, enteritis, flatulence, gastritis, intestinal colic, and spasms affecting the bile ducts, gallbladder, and digestive system [44]. *Cinnamomum zeylanicum* (cinnamon) follows with an FL value of 85%, highlighting its notable role in digestive health. Numerous studies worldwide have documented cinnamon’s medicinal efficacy in addressing digestive diseases [45]. Other species, such as *Camellia sinensis* (76%) and *Tripleurospermum disciforme* (62%), also hold significant relevance but exhibit slightly more diversified uses. For instance, *Tripleurospermum* species are applied in treating digestive, skin, and respiratory disorders [13]. Traditional medicine also values *Camellia sinensis* L. (tea) for its effectiveness in managing digestive issues, including its use in alleviating bloody diarrhea [46].

*Tripleurospermum disciforme* and *Thymus vulgaris* exhibit FL values of 37% and 30%, respectively, in the treatment of dermatological conditions, suggesting their broader medicinal roles beyond skin-related disorders. Additional ethnobotanical evidence from Lorestan Province, Iran, confirms that *Thymus daenensis*, *Thymus eriocalyx*, *Mentha spicata*, and *Mentha longifolia* are traditionally employed as gastronomics and for managing skin disorders [43]. Notably, *Tripleurospermum disciforme* (C.A.Mey.) Schultz has traditionally been used for the treatment of prostate disorders, kidney stones, stomach aches, and hypoglycemia. Similarly, *Thymus lancifolius* Celak has been applied in managing stomach aches, hypothermia, diarrhea, flatulence, and gastric ulcers. Moreover, the therapeutic potential of *Mentha spicata* L. and *Mentha longifolia* (L.) Hudson has been validated for their effectiveness in alleviating stomach aches, vomiting, food poisoning, fever, diarrhea, infections, and even snake bites [47].

Of particular interest is *Thymus kotschyanus*, which demonstrates a higher FL value of 50% specifically for wound healing, emphasizing its specialized role in tissue regeneration. This therapeutic potential is attributed to its high concentrations of thymol and carvacrol, phenolic compounds renowned for their antibacterial and anti-ulcer properties [48,49,50].

In the domain of gastrointestinal health, *Echium amoenum* presents a relatively modest FL value of 25%, reflecting its more targeted, albeit limited, use for digestive complaints. This aligns with findings from Alamout, Qazvin, where its application for stomach-related issues is well documented [51]. Likewise, *Stachys lavandulifolia* is traditionally utilized for ailments such as headaches, hypothermia, hyperlipidemia, hypoglycemia, gastric ulcers, and infections [47,51,52]. Furthermore, an ethnobotanical study in North Khorasan identified *Echium amoenum* Fisch. & C.A.Mey, *Tripleurospermum disciforme* (C.A.Mey.), and *Stachys turcomanica* Trautv. as the most frequently cited sedative agents [34].

*Crocus sativus* L. (saffron) stands out with a notable FL value of 73% for cardiac disorders. The therapeutic efficacy of its petal extract is largely due to bioactive flavonoids such as kaempferol and crocin, which confer significant antioxidant properties that combat lipid peroxidation, thereby contributing to the prevention and management of atherosclerosis and cardiovascular diseases [53,54,55]. Additionally, *Crocus sativus* L. has demonstrated considerable effectiveness in the treatment of depressive disorders [56,57].

Finally, *Zingiber officinale* (ginger) emerges as a leading traditional remedy for respiratory ailments, boasting the highest FL value of 77%. It is extensively used for managing conditions such as coughs, colds, influenza, sore throats, and throat infections, underscoring its prominence in ethnomedicinal practices [58,59].

Table 3 highlights the importance and utilization of various medicinal plant species by presenting the number of participants who cited each species, along with key metrics such as Relative Frequency of Citation (RFC) and Use Value (UV). These indices, commonly used in ethnobotanical studies, help to measure the cultural significance and practical applications of plant species in traditional medicine.

The most frequently cited species in the dataset were *Tripleurospermum disciforme* and *Thymus kotschyanus*, referred by 42 and 40 participants, respectively, with high RFC values. Their UVs, 0.42 and 0.4, respectively, suggest that they are not only culturally significant, but also frequently utilized for medicinal purposes. These findings indicate a high level of familiarity and reliance on these plants in the community. Other species such as *Echium amoenum* (32 participants, RFC 0.32, UV 1.31) and *Mentha* spp. (30 participants, RFC 0.3, UV 1) are also notable. They reflect a moderate level of both recognition and practical use in traditional applications. These plants may play a role in treating common ailments, thus maintaining a prominent place in the ethnobotanical landscape.

Our findings align with previous ethnobotanical investigations conducted in Bojnord, North Khorasan Province, Iran, where *Tripleurospermum disciforme* (C.A.Mey.) Sch.Bip. and *Echium amoenum* Fisch. & C.A.Mey. were also cited with notably high RFC, reflecting their prevalent use in local traditional medicine. Specifically, *Tripleurospermum disciforme* (C.A.Mey.) Sch.Bip. was associated with a substantial number of use reports [34]. Similarly, in the Central Zagros region of Lorestan Province, species such as *Mentha longifolia*, *Mentha spicata*, *Mentha piperita*, *Thymus daenensis*, and *Thymus eriocalyx* exhibited high RFC values, suggesting widespread therapeutic application. The FL for *Tripleurospermum disciforme* (C.A.Mey.) Sch.Bip. (BabunehDashti) has been reported at 58 [43], underscoring its therapeutic relevance. Furthermore, in Hamedan Province, Iran, *T. disciforme* (C.A.Mey.) Sch.Bip. has been traditionally utilized for the treatment of kidney stones. Additional species, including *Thymus lancifolius* Celak and *Achillea biebersteinii*, have been employed in ethnoveterinary medicine, particularly for managing digestive disorders [47]. *Thymus lancifolius* Celak has also been verified for its effectiveness in treating hypothermia, stomach aches, and diarrhea, as well as for its carminative and anti-ulcer properties [47].

*Zingiber officinale* Roscoe is traditionally recognized for its role in treating vomiting, indigestion, colds, and musculoskeletal pain [60]. It also possesses well-documented anti-inflammatory and antioxidant properties [61], in addition to demonstrated glucose and lipid lowering effects, contributing to its potential role in obesity management [62]. Its ethnomedical significance extends beyond Iran; for instance, it is used in Ghana and Uganda for treating tuberculosis [63,64], and in India it is employed as an antiemetic and for its anticancer properties [65].

The UV metric, which indicates the relative importance of a plant based on the frequency of its reported use by informants, further supports these findings. The highest UVs were recorded for *Tripleurospermum disciforme* (1.72), *Thymus kotschyanus* (1.75), and *Echium amoenum* (1.31), aligning well with their RFC scores. Interestingly, several species with lower citation frequencies, such as *Crocus sativus* (saffron), *Camellia sinensis*, *Rosmarinus* spp., *Cinnamomum zeylanicum*, *Lavandula angustifolia*, *Stachys lavandulifolia*, *Malva sylvestris*, and *Zingiber officinale*, still exhibited a UV of 1. This suggests that while their use may be more specialized or context-specific, they nonetheless hold significant medicinal value.

Previous studies have validated the therapeutic potential of *Lavendula angustifolium* (lavender), citing its effectiveness as a sedative and in the treatment of nervousness, insomnia, circulatory disorders, dyspeptic symptoms, and even aggression reduction [56,66,67]. Of the 36 plant species identified in the present study, many have also been reported across various regions of Iran. These include *Lavandula angustifolia* Mill., *Camellia sinensis* L., *Salix aegyptiaca* L. *Salix aegyptiaca* Var. Lonigifron, *Stachys lavandulifolia* Vahl., *Citrus aurantium* L., *Cinnamomum zeylanicum* Nees., *Rosmarinus officinalis*, *Mentha pulegium* L., *Mentha longifolia* (L.) Hudson, *Mentha piperita* L., *Mentha aquatica* L., *Mentha spicata*, *Crocus sativus* L., *Echium amoenum* Fisch. & C.A.Mey., *Tripleurospermum disciforme* (C.A.Mey.) Sch.Bip, *Tripleurospermum disciforme* L., *Rosmarinus officinalis* L., *Rosa x damascena* Mill., *Thymus vulgaris* L., *Thymus daenensis*, *Thymus eriocalyx*, *Thymus lancifolius* Celak., *Adiantum capillus*-*veneris* L., *Salvia sclarea* L., *Malva sylvestris* L., *Achillea santolinoides* subsp. *Wilhelmsii* (K.Koch) Greuter., *Achillea millefolium* L., *Achillea biebersteinii* Afan., *Achillea wilhelmsii* C. Koch., *Achillea* vermicularis Trin, *Urtica urens* L., *Ziziphora clinopodioides* Lam., *Salvia officinalis* (sag), and *Camellia sinensis* [34,35,43,47,51,56,68,69].

### 2.2. Tripleurospermum Disciforme

Among the medicinal plants documented in the ethnobotanical survey, *Tripleurospermum disciforme* emerged as the most frequently cited species, recording the highest relative frequency of citation (RFC) and use value (UV). These indices reflect both widespread community reliance and strong consensus regarding its therapeutic effectiveness in regions of Iran, particularly in the management of digestive, respiratory, skin, and nervous system disorders, thereby justifying its selection for further scientific exploration. The subsequent chemical and biological analyses provide initial experimental support for its traditional uses, particularly in gastrointestinal health, and demonstrate how ethnobotanical knowledge can effectively guide pharmacological investigations.

#### 2.2.1. Phytochemical Characterization

The untargeted metabolomic profiling of *Tripleurospermum disciforme* (C.A.Mey.) Sch.Bip extracts revealed a diverse array of secondary metabolites. A total of 43 features were annotated based on a combination of spectral library matching (SL), an in-house analyte list of phenolic compounds (AL), and SmartFormula (SF) calculations (Table 4). These annotations were supported by high-resolution mass spectrometric data, including accurate *m*/*z* values, retention times, collisional cross section (CCS) values, and MS/MS fragmentation patterns. Notably, several phenolic compounds such as neochlorogenic acid, caffeic acid, and p-HPEA-AC (*p*-hydroxyphenylethanol acetate) were recurrently detected across extracts.

Differences in the phytochemical profiles were evident depending on the solvent used, with methanol and ethyl acetate showing greater extractive efficiency compared to hexane, particularly for polar and mid-polar metabolites. These findings highlight the importance of solvent selection in non-targeted metabolomic studies and reflect the chemical diversity of the extracts.

The annotation strategy, integrating accurate mass, isotopic pattern, MS/MS fragmentation, and ion mobility data, aligns with current standards in untargeted metabolomics and offers a high degree of confidence for structural assignment. Although further validation through analytical standards or orthogonal techniques could complement the results, the adopted workflow provides a comprehensive and reliable overview of the metabolite composition of *Tripleurospermum disciforme* (C.A.Mey.) Sch.Bip. extracts.

**Figure 4:**
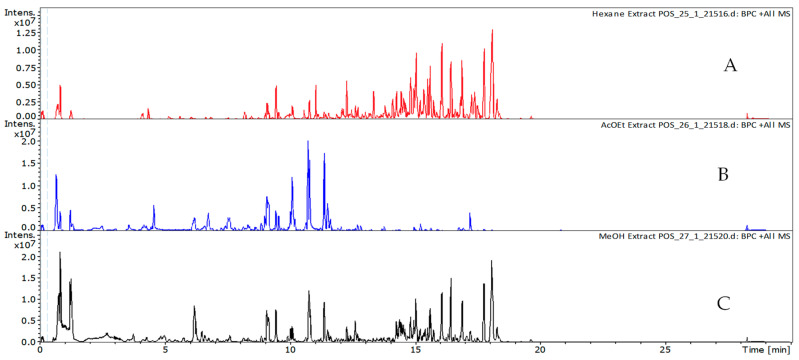
Base peak chromatograms (BPCs) of the extracts in positive ionization mode: (**A**) hexane, (**B**) ethyl acetate, and (**C**) methanol.

**Figure 5:**
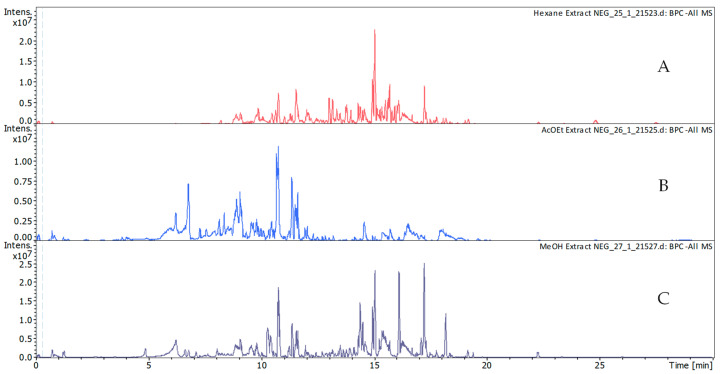
Base peak chromatograms (BPCs) of the extracts in negative ionization mode: (**A**) hexane, (**B**) ethyl acetate, and (**C**) methanol.

A detailed list of the annotated compounds, along with their chromatographic, spectral, and ion mobility properties, is available in Supplementary Table S1. In the this table, some compounds still appear more than once (e.g., 4-(4-hydroxyphenyl)-2-butanone, scutellarein, neochlorogenic acid), which reflects differences in retention times across extracts and/or the presence of isomeric forms. CCS values were used to support this interpretation, since CCS differences of ≤2% are generally attributed to experimental variability, while differences above 3% combined with distinct retention times strongly suggest the occurrence of isomers. Figure 4 and Figure 5 show the base peak chromatograms (BPCs) obtained in positive (A–C) and negative (D–F) ionization modes for the hexane (A), ethyl acetate (B), and methanol (C) extracts.

#### 2.2.2. Antioxidant Activity

Among the evaluated extracts of *Tripleurospermum disciforme* (C.A.Mey.) Sch.Bip., the ethyl acetate extract exhibited the most potent antioxidant activity, as evidenced by its IC_50_ value of 12.496 ± 4.153 μg/mL in the DPPH free radical scavenging assay, while the methanol and chloroform extracts (which came from successive extractions) displayed moderate activity compared to the gallic acid standard. Notably, all extracts, except for the hexane extract (AAI < 0.5), demonstrated antioxidant activity ranging from moderate to strong (Table 5).

In contrast, recent studies [70] reported that the methanol extract of Tripleurospermum limosum (Maxim.) Pobed (which was a first extraction) exhibited strong antioxidant activity (IC_50_ = 0.0304 mg/mL) for DPPH assay, but also across various assays, including ABTS, hydroxyl, and superoxide radical scavenging tests. It is important to highlight that numerous studies have emphasized the critical role of solvent selection in extracting phenolic compounds. Water and aqueous methanol are particularly effective solvents for isolating phenolic compounds from plants in the genus Tripleurospermum, including *Tripleurospermum insularum* Inceer & Hayırlıoglu-Ayaz [71] and *Tripleurospermum disciforme* (wild chamomile) [72], as well as for extracting flavonoids from *Tripleurospermum disciforme* [20]. The antioxidant activity observed in *Tripleurospermum disciforme* aligns with findings for the methanolic extracts of *Tripleurospermum insularum* [71] and Tripleurospermum limosum [70], both of which exhibit notable DPPH scavenging capabilities. Additionally, research on the Asteraceae family has identified the presence of significant caffeoyl derivatives in *Tripleurospermum disciforme* [22], Tripleurospermum oreades (Boiss.) Rech.f. [73], and *Tripleurospermum insularum* Inceer & Hayırlıoglu-Ayaz [71], which are recognized for their robust antioxidant properties [74].

Here, considering the very strong antioxidant activity of ethyl acetate extract, it can be suggested that chlorogenic acid may play a role in this activity, due to its absence in other extracts. Chlorogenic acid (5-O-caffeoylquinic acid) is a phenolic compound belonging to the hydroxycinnamic acid family. It is known for its wide range of beneficial health effects, including managing metabolic syndrome, antidiabetic and anti-inflammatory activity, lipid-lowering and blood pressure-regulating properties, and predominantly significant antioxidant activity [75,76,77,78,79]. When evaluating antioxidant activity and correlating it with plant composition, multiple factors can influence the results, including seasonal and geographic variation, which directly affect phytochemical profiles. Notably, cooler temperatures prevailing at higher elevations act as a key ecological driver, stimulating the production of antioxidant compounds regardless of interspecific genetic variation [13,80]. Consistently, numerous studies have shown that plants adapted to high-altitude environments tend to accumulate greater amounts of phenolic constituents compared to those growing at lower elevations [81,82,83].

### 2.3. Antimicrobial Activity

In the disc diffusion assay (Supplementary Table S2), limited antimicrobial activity was observed for the tested fractions. The most notable effect was found in the ethyl acetate extract, which exhibited inhibitory activity against Staphylococcus aureus and Bacillus cereus, with inhibition zones of 11.3 mm and 12 mm, respectively. Additionally, the chloroform extract showed inhibitory activity against B. cereus, with an inhibition zone of 10 mm. In contrast, the methanol and hexane fractions displayed low to no antimicrobial activity against the tested bacterial strains.

Regarding MIC determination (Table 6), the strongest activity was observed against *B. cereus*, with MIC values ranging from 2500 to 312 µg/mL for *T. disciforme* extracts. *S. aureus* also exhibited some susceptibility, though at higher MIC values of 5000 and 2500 µg/mL. These findings provide initial evidence for the antimicrobial potential of *T. disciforme* and lay the groundwork for future studies aimed at identifying specific bioactive compounds and exploring a broader spectrum of microbial targets, thereby linking ethnobotanical knowledge with pharmacological evaluation.

As found in this study, previous studies showed that *T. disciforme* collected from Tehran, Guilan, and Fars provinces has demonstrated low antibacterial activity in methanol extracts, with inhibition zones of 12 mm and 14 mm against *Staphylococcus epidermidis* and *S. aureus*, respectively, at high concentrations of extracts (64 mg/mL) [20]. However, Tofighi et al. (2015) reported no antibacterial activity of *T. disciforme* methanolic extracts against *Escherichia coli*, *Pseudomonas aeruginosa*, *Candida albicans*, *Aspergillus niger, Bacillus cereus*, and *Bacillus subtilis* [20]. Derivatives of 4-hydroxycoumarin have been shown to possess activity against *Bacillus cereus* and *Bacillus subtilis* [84,85,86]. Similarly, chlorogenic acid (5-*O*-caffeoylquinic acid) exhibits activity against the *Bacillus* genus [87,88], with the study by Wu et al. (2020) [89] demonstrating that the chlorogenic acid caused a notable reduction in intracellular adenosine triphosphate (ATP) levels, possibly by affecting the material and energy metabolism or interfering with cellular signal transduction pathways. Additionally, it exerted a bacteriostatic effect by disturbing the intracellular metabolic balance of the tricarboxylic acid (TCA) cycle and glycolytic pathway, ultimately resulting in metabolic dysfunction and the death of *B. subtilis* [89]. Therefore, the presence of these compounds in the ethyl acetate extract may support the antimicrobial activity of this extract.

In turn, several studies have highlighted the strong antimicrobial properties of essential oils derived from various plants, including *T. disciforme* (C.A.Mey.) Sch.Bip, which achieved a MIC of 250 μg/mL against *S. aureus* or of 125 μg/mL against *B. subtilis*, *Klebsiella pneumoniae*, and *E. coli* and 62.5 μg/mL against *P. aeruginosa* [90]. Based on the evidence obtained from this study, we conclude that *T. disciforme* extracts exhibit weaker antibacterial properties compared to its essential oil, indicating that the antibacterial potential of *T. disciforme* resides primarily in volatile compounds rather than its crude fractions. It is well-established that the chemical composition of plants is influenced by various factors such as species, solvent type, extraction method, and ecological conditions, all of which subsequently affect their antimicrobial efficacy [91].

However, further studies are required to explore its full therapeutic potential and to identify the specific bioactive compounds responsible for its antimicrobial properties.

### 2.4. Cellular Viability

**Figure 6:**
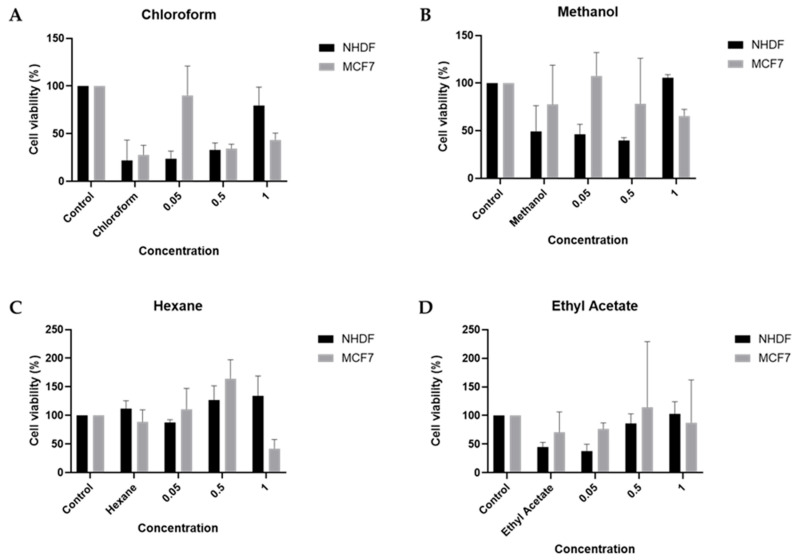
Effect of *Tripleurospermum disciforme* (C.A.Mey.) Sch.Bip. extracts on the viability of NHDF and MCF-7 after 48 h exposure. Extracts were obtained using four solvents, (**A**) chloroform, (**B**) methanol, (**C**) hexane, and (**D**) ethyl acetate, and tested at three concentrations (0.05, 0.5, and 1 mg/mL). For internal conditions, Control refers to cells with medium, followed by the solvent controls (20 µL of the corresponding solvent, equivalent to the highest volume present in the treatment wells).

The effect of *T. disciforme* (C.A.Mey.) Sch.Bip. extracts on cell viability was assessed in two distinct human cell lines: NHDF (normal human dermal fibroblasts) and MCF-7 (breast adenocarcinoma). Extracts were obtained using four different solvents, chloroform, methanol, ethyl acetate, and hexane, applied at increasing concentrations (0.05%, 0.5%, and 1% *v*/*v*). Cell viability was quantified using the 3-(4,5-dimethylthiazol-2-yl)-2,5-diphenyltetrazolium bromide (MTT) assay and normalized to total protein content, allowing for the correction of differences in cell density or growth rates (Figure 6).

In NHDF cells, the chloroform and methanol extracts displayed similar patterns: both the solvent controls and the lowest extract concentrations (0.05 and 0.5 mg/mL) significantly reduced cell viability when compared to the untreated control. Specifically, chloroform alone reduced viability (*p* = 0.0003 vs. control), as did the 0.05 mg/mL (*p* = 0.0004) and 0.5 mg/mL (*p* = 0.0010) concentrations. However, at 1 mg/mL, a significant increase in viability was observed compared to the chloroform-only control (*p* = 0.0031) and to lower concentrations (*p* = 0.0132 vs. 0.5 mg/mL), although not significantly different from the untreated control (*p* = 0.42). A comparable trend was seen with the methanol extract: methanol alone reduced viability (*p* = 0.0056), as did 0.05 mg/mL (*p* = 0.0037) and 0.5 mg/mL (*p* = 0.0016), while the highest concentration significantly improved viability (*p* = 0.0026 vs. methanol control). Regarding ethyl acetate, the concentrations 0.5 and 1 significantly increased viability when compared to solvent-only control, at *p* = 0.028 and *p* = 0.0031, respectively. In contrast, the hexane extract did not produce any statistically significant changes in NHDF viability across all tested concentrations, despite an apparent upward trend in cell viability at higher doses.

In MCF-7 cells, a markedly different response profile was observed. The chloroform extract induced a strong cytotoxic effect: chloroform alone significantly reduced viability (*p* = 0.0011 vs. control), and both 0.5 mg/mL (*p* = 0.0024) and 1 mg/mL (*p* = 0.0067) concentrations maintained this reduction. Interestingly, the 0.05 mg/mL extract concentration increased viability compared to chloroform-only (*p* = 0.0034), but remained below control, suggesting a biphasic response.

In contrast, the methanol and ethyl acetate extracts produced no statistically significant effects on MCF-7 viability at any concentration. Although fluctuations were noted, particularly at 0.5 mg/mL ethyl acetate, where a slight increase in viability was observed, the high variability and lack of significance indicate minimal impact on tumour cell metabolism or survival.

The hexane extract, however, induced this effect in MCF-7 cells. At 0.5 mg/mL, viability was significantly increased (*p* = 0.027 vs. hexane-only). In contrast, the 1 mg/mL concentration caused a significant reduction in viability (*p* = 0.0010 when compared to 0.5 mg/mL), suggesting dose-dependent cytotoxicity after initial stimulation.

Collectively, these findings indicate that *T. disciforme* (C.A.Mey.) Sch.Bip. contains biologically active compounds capable of modulating cell viability in a solvent-concentration dependent manner, with clear differences in sensitivity between normal and tumoral cells. While NHDF cells exhibited cytoprotective responses at higher extract concentrations (particularly with chloroform and methanol), MCF-7 cells were more susceptible to cytotoxic effects, especially with the chloroform and hexane extracts. In the majority of the concentrations, for all solvents, there is no tumour cell proliferation, suggesting a favourable profile for further investigation.

These results highlight the potential of *T. disciforme* (C.A.Mey.) Sch.Bip. as a source of bioactive molecules with selective effects on human cells. The extract concentrations that enhanced viability in NHDF cells did not promote proliferation in tumour cells, and in some cases induced cytotoxicity. Further studies are needed to clarify these differential effects and to explore their potential applications in cytoprotection or anticancer therapy.

## 3. Materials and Methods

### 3.1. Ethnobotanical Surveys

#### 3.1.1. Guilan, Qazvin, and Alborz Provinces Geo-Ethnographical Profile

Guilan Province is located in the north and northwest of Iran, covering an area of 14,711 km^2^. It lies between latitudes 36°33′ to 38°27′ N and longitudes 48°32′ to 50°36′ E. The province is administratively divided into 16 counties, 52 cities, 43 districts, 109 rural areas, and 2583 villages, with a total population of 2,530,686. Geographically, Guilan Province is positioned south of the Caspian Sea, boasting approximately 300 km of coastline, and north of the Alborz Mountains, where the average elevation reaches 3000 m. Due to its unique topographical and climatic conditions, Guilan is recognized as one of the wettest provinces in Iran [92].

The northern provinces of the country Guilan, Mazandaran, and Golestan, as well as the Talesh region in Azerbaijan, are home to 3234 vascular plant species, encompassing 856 genera and 148 families. Of these, 475 species of medicinal plants have been identified in the forests of Guilan Province [93]. The Hyrcanian forests, which closely resemble the forests of Central Europe, are situated in the northern provinces of Iran Guilan, Mazandaran, and Golestan. Stretching 800 km along the western Caspian Sea and the northern Alborz Mountain Range, these forests cover an area of 1.84 Mha and are 110 km wide [94].

The population of Qazvin Province was approximately 1.3 million, with 600,000 residing in Qazvin City. Despite comprising only 1% of Iran’s total land area, it is in the northern half of the country, between latitudes 35°37′ and 36°45′ N and longitudes 48°45′ and 50°50′ E. Although it ranks as the 26th largest province in Iran, Qazvin boasts a remarkably diverse climate due to its position between the arid central plateau and the northeastern mountainous regions. The province also exhibits significant altitudinal variation, with Siyalan Mountain reaching 4175 m as its highest point and the banks of Manjil Dam Lake at 300 m as its lowest. This elevation disparity greatly influences both precipitation and temperature. Annual rainfall varies from 210 mm in the eastern regions to 550 mm in the northwest, while temperature fluctuations are considerable, with extreme highs recorded in the central plains and severe lows in the northwestern highlands of Avaj. Average temperatures gradually decrease from the lowlands toward the foothills and mountainous areas [95].

Alborz Province, covering 519,391.5 ha, constitutes approximately 0.3% of Iran’s total land area. Geographically, it lies between 50° and 51.30° east longitude and 30.35° and 30.36° north latitude, positioned along the southern slopes of the Alborz mountain range. The province exhibits diverse topographical characteristics. Its northern areas ascend to altitudes of 4104 m, forming a continuous mountain range stretching from east to west. Meanwhile, the central region consists primarily of plains, sitting at an average altitude of approximately 1500 m. Overall, most of the province lies at elevations ranging from 1500 to 2000 m [96].

#### 3.1.2. Field Interview

This survey was conducted over a six-month period during the first semester of 2023. A semi-structured, face-to-face questionnaire was administered to 100 local individuals in the herbal markets of the Guilan, Qazvin, and Alborz provinces. The questionnaire primarily focused on ethnobotanical claims and traditional beliefs held by local communities in these three regions. It also gathered demographic and personal information, including respondents’ age, gender, relationship with the locality, education level, knowledge level, most used plants, plant identification, utilized plant parts, and methods of preparation.

Subsequently, scientific identification and authentication of the cited plants were carried out using Flora Iranica [97], Flora of Iran [98], and various herbal literature sources [6,69]. Additionally, the accuracy of the scientific names of the medicinal plants was verified through www.theplantlist.org.

#### 3.1.3. Quantitative Analysis of the Ethnobotanical Results

All statistical results were firstly prepared using Microsoft Excel 2013 (Windows, Washington, DC, USA).

Informant Consensus Factor (ICF) was utilized to assess the consistency of traditional knowledge regarding plant usage across various ailment categories, using Equation (1)

ICF = (N_ur_ − N_t_)/(N_ur_ − 1) (1)

where N_ur_ is the total number of use-reports for a specific illness category, and N_t_ is the number of taxa utilized in that category. The ICF, which ranges from 0 to 1, reflects the degree of agreement among informants. A higher ICF value suggests a concentrated reliance on a few specific species, possibly indicating effective knowledge transmission or a shared cultural understanding. In contrast, lower values imply inconsistency in plant selection, potentially due to sporadic knowledge sharing or arbitrary choices by informants [26,41].

Fidelity level (FL) index was assessed to identify the most culturally significant plant species associated with specific health conditions, especially in cases where multiple species are used for the same ailment. The FL was calculated using Equation (2)

FL = (Np/N × 100)(2)

where Np is the number of participants who cited a plant for a particular disease, and N is the total number of participants who reported that plant for any ailment. Higher FL percentages indicate a stronger preference and consensus on using a given plant species for a specific health issue, by the participants of the study area [26,41].

Relative Frequency of Citation (RFC) index does not differentiate between specific uses or categories of use for a plant; it is simply a general record of how often a plant is cited in relation to any reported use by an individual. This means that it reflects the frequency with which a plant is mentioned in ethnobotanical studies, but it does not provide detailed information about the specific purpose or category (e.g., medicinal, culinary) for which the plant is used. The RFC index is expressed by Equation (3)

RFC = FC/N (3)

where FC is the number of informants who mentioned a particular plant frequency of citation, and N is the total number of informants in the survey. The index varies between 0 (when no one considers a plant to be useful) and 1 (if all the participants mention a plant as useful). Unlike other indices, RFC index does not account for the category of use or the number of different uses reported [26,41].

Use value (UV) index was calculated using Equation (4) to gauge the overall significance of each plant species in the local traditional knowledge system, i.e.,

UV = ΣUi/N(4)

where Ui represents the number of different uses cited by each informant for a species, and N is the total number of informants in the survey. A higher UV indicates a broader or more frequent application of a plant in ethnomedicinal practices [26,41].

### 3.2. Collection and Identification of Plant Materials

The dried flowering tops of *Tripleurospermum disciforme* were sourced from a local market in Guilan, Iran. A voucher specimen was subsequently deposited in the herbarium of Guilan University of Medical Sciences.

### 3.3. Extraction (Preparation of Extracts)

About 1500 g of the top flowers of *T. disciforme* was extracted via the percolation method by methanol at room temperature for 24, 48, and 72 h. The extracts were concentrated using a rotary evaporation system. The crude extract of *T. disciforme* was re-extracted with increasing polarity solvents, n-hexane, chloroform, ethyl acetate, and methanol, respectively, using an ultrasound bath for 20 min, three times, with frequent shaking at 40 °C. The extracts were concentrated using the same rotary evaporation system, weighed, and stored in 4 °C, until phytochemical characterization and analysis of the biological activities.

### 3.4. Phytochemical Characterization and Phenolic Profile

An untargeted metabolomic approach was employed to characterize the phytochemical composition of the samples. Analyses were conducted by ultra-high performance liquid chromatography (UHPLC) coupled to a trapped ion mobility time-of-flight mass spectrometer (timsTOF-MS; Bruker Daltonics, Bremen, Germany), equipped with a VIP-HESI electrospray ionization source. Samples were previously dissolved in minimal volumes of hexane, ethyl acetate, or methanol, and 5 µL of each solution was injected into a ZORBAX Eclipse XDB-C18 RRHD column (2.1 × 100 mm, 1.8 µm; Agilent Technologies, Santa Clara, CA, USA). Chromatographic separation was achieved using a binary mobile phase consisting of 0.1% formic acid in water (solvent A) and 0.1% formic acid in acetonitrile (solvent B), under a flow rate of 0.4 mL/min. The gradient programme lasted 30 min, including a re-equilibration phase.

Mass spectrometric detection was performed in both positive and negative ionization modes. The capillary voltage was set at ±4500 V and the end plate was offset at ±500 V. Nitrogen was used as the nebulising gas (8 bar), drying gas (8 L/min at 240 °C), and sheath gas (4 L/min at 450 °C). Spectra were acquired over an *m*/*z* range of 20–1300 in both MS and MS/MS mode using Parallel Accumulation–Serial Fragmentation (PASEF). Ion mobility separation was recorded over a 1/K_0_ range of 0.45–1.45 V·s/cm^2^ with a ramp time of 100 ms.

Data processing was carried out using *Data Analysis* (v6.1) and *MetaboScape* (v7.0.1) software from Bruker. Spectral features (“buckets”) were extracted based on *m*/*z*, retention time, and collisional cross section (CCS), applying an intensity threshold of 10,000 counts. Compound annotation was performed using three strategies: (i) spectral library matching (SL), (ii) an in-house analyte list (AL) of phenolic compounds (name and molecular formula only), and (iii) SmartFormula (SF) calculations restricted to CHNOPS atom sets.

Combining these approaches, a total of 43 tentative identifications were obtained through SL + AL + SF. Additional matches were retrieved using AL + SF (237 features), SL + SF (64 features), and SF alone (1605 features).

### 3.5. Biological Activities Evaluation

#### 3.5.1. Antioxidant Activity

The antioxidant activity was evaluated using the 2,2-diphenyl-1-picrylhydrazyl (DPPH) free radical scavenging assay. A calibration curve was constructed with methanol solutions of DPPH (85–4.25 mg/L). Absorbance was measured at 517 nm, and the capacity of free radical scavenging was calculated accordingly to Luís et al. [99]. The IC_50_ was calculated graphically using the extract concentration versus the corresponding radical scavenging capacity. The antioxidant activity was expressed through the antioxidant activity index (AAI), calculated as follows: AAI = (final concentration of DPPH in the control)/(IC50). The AAI allowed for the classification of the antioxidant activity of the samples as poor (AAI ≤ 0.5), moderate (0.5 < AAI ≤ 1.0), strong (1.0 < AAI < 2.0), or very strong (AAI ≥ 2.0) [99,100].

#### 3.5.2. Antimicrobial Activity

##### Microorganisms and Media

Hexane, chloroform, ethyl acetate, and methanol extracts were tested against three Gram-positive (*Staphylococcus aureus* ATCC 25923, *Enterococcus faecalis* ATCC 29212, and *Bacillus cereus* ATCC 11778) and five Gram-negative (*Pseudomonas aeruginosa* ATCC 27853, *Salmonella* Typhimurium ATCC 13311, *Escherichia coli* ATCC 25922, *Klebsiella pneumoniae* ATCC 13883, *Acinetobacter baumannii* LMG 1025) bacteria. All the strains were cultured on Brain Heart Infusion Agar (BHIA) and incubated at 37 °C for 24 h before any antimicrobial assay.

##### Determination of Disc Diffusion Assay

The antimicrobial activity of *Tripleurospermum disciforme* (C.A.Mey.) Sch.Bip was evaluated by disc diffusion assay, following the M2-A8 method as described by the Clinical Laboratory and Standards Institute (CLSI) for bacteria. Bacteria inocula were prepared by the direct suspension of colonies in saline solution (NaCl 0.85% (*w*/*v*)) and adjusted to 0.5 McFarland (about 1 to 2 × 10^8^ (CFU/mL)). Discs with a diameter of 6 mm were each impregnated with 20 µL of each extract dissolved in dimethylsulfoxide (DMSO; Scharlab, Spain) at a concentration of 10 mg/mL. For the negative control, DMSO was used instead. Then, the Müeller–Hinton Agar (MHA) (Liofilchem, Italy) plates were inoculated with the cellular suspension, and the discs previously prepared were placed over the agar. The plates were incubated at 37 °C for 24 h. Following incubation, all the plates were visually checked for inhibition zones, and the diameters were measured in mm. Each experiment was performed three independent times.

##### Determination of Minimum Inhibitory Concentration (MIC)

The MIC of the extracts was evaluated by the microdilution method as described by [101]. Serial dilutions of each of extract (from 5000 µg/mL to 39 µg/mL) were prepared in a 96-well plate (50 µL per well) in Müeller–Hinton Broth (MHB) (Liofilchem, Italy). The cellular suspension was prepared as described above, diluted in MHB, and added to wells to obtain a final cellular concentration of 5 × 10^5^ CFU/mL in each well. A positive control was used containing tetracycline. Wells without the tested plant extracts were used as a growth control, and wells without bacteria were used as a negative growth control. The plate was incubated for 24 h at 37 °C; after that, 30 µL of resazurin (0.1%) was added in each well of the microtiter plate and incubated at 37 °C for 2 h. No color change (blue) indicated inhibition of the tested bacterium. The MIC was considered the lowest concentration of the extract that inhibited bacterial growth. Each assay was performed at least three independent times.

### 3.6. In Vitro Studies Using NHDF and MCF-7 Cells

#### 3.6.1. Cell Culture

Human breast adenocarcinoma cells (MCF-7; ATCC^®^ HTB-22™) and normal human dermal fibroblasts (NHDF; ATCC^®^ PCS-201-012™) were obtained from the American Type Culture Collection (ATCC, Manassas, VA, USA). Unless otherwise specified, all chemicals (analytical grade), reagents, culture media, and supplements were purchased from Sigma-Aldrich (St. Louis, MO, USA).

Cells were routinely maintained in 75 cm^2^ culture flasks at 37 °C in a humidified atmosphere containing 5% CO_2_. NHDF cells were cultured in RPMI-1640 medium supplemented with 10% fetal bovine serum (FBS), L-glutamine (0.02 M), HEPES (0.01 M), sodium pyruvate (0.001 M), and 1% antibiotic/antimycotic (10,000 U/mL penicillin, 10 mg/mL streptomycin, and 25 mg/mL amphotericin B). The culture medium was renewed every 48 h until cells reached approximately 90–95% confluence. MCF-7 cells were maintained in Dulbecco’s Modified Eagle Medium (DMEM, high glucose) supplemented with 10% FBS and 1% antibiotic/antimycotic.

At confluence, adherent cells were detached using trypsin-EDTA solution (0.125 g/L trypsin and 0.02 g/L EDTA). Prior to each experiment, cell viability was assessed by trypan blue exclusion using a Neubauer haemocytometer, and suspensions were adjusted to the required density in the appropriate complete culture medium.

#### 3.6.2. Preparation of the Solutions of the Compounds Under Study

Hexane, chloroform, methanol, and ethyl acetate extracts of *T. disciforme* (C.A.Mey.) Sch.Bip. extract were dissolved at a final concentration of 10 mg/mL in their corresponding solvents. The mixtures were sonicated for approximately 3 h at 40 °C, filtered, and the resulting supernatant was stored at 4 °C.

#### 3.6.3. Cytotoxicity Assay and Protein Quantification

NHDF and MCF-7 cells were seeded at a density of 2 × 10^4^ cells/mL in a 96-well plate (200 μL/well). Two plates were prepared for each cell type. After incubation for 48 h at 37 °C in a humidified atmosphere containing 5% CO_2_, cell adherence was verified and culture medium was replaced with treatment solutions.

Cells were exposed for 48 h to *T. disciforme* (C.A.Mey.) Sch.Bip. extracts (hexane, chloroform, methanol, or ethyl acetate) diluted in complete culture medium at final concentrations of 0.05, 0.5, and 1 mg/mL. Negative controls included untreated cells, while solvent controls received 20 µL of the corresponding solvent, equivalent to the highest volume present in the treatment wells. All experiments were conducted in triplicate and independently repeated at least twice.

In one plate per cell type, antiproliferative activity was assessed using the MTT assay. Following 48 h exposure, the medium was aspirated and cells were washed with 100 µL phosphate-buffered saline (PBS). MTT solution (5 mg/mL in PBS) was added to each well and incubated for 4 h at 37 °C. After removal of the MTT solution, formazan crystals were solubilised in dimethyl sulfoxide (DMSO; Sigma-Aldrich, St. Louis, MO, USA). Absorbance was measured at 570 nm using an xMark™ microplate spectrophotometer (Bio-Rad Laboratories, Hercules, CA, USA).

In parallel plates, total protein content was quantified using the Pierce™ BCA Protein Assay Kit (Thermo Scientific, Waltham, MA, USA, cat. no. 23227), according to the manufacturer’s instructions. Briefly, after treatment, cells were washed with PBS, and plates were wrapped in aluminum foil and frozen at −20 °C for at least 24 h. The working reagent was prepared by mixing 50 parts of Reagent A with 1 part of Reagent B. Plates were thawed, and 20 µL of PBS was added to each well, followed by 200 µL of working reagent. Plates were mixed on a shaker for 30 s and incubated at 37 °C for 30 min. After cooling to room temperature, absorbance was recorded at 562 nm, and protein concentration was determined against a standard curve.

#### 3.6.4. Statistical Analysis

All statistical analyses were performed using GraphPad Prism version 10.6.0 (GraphPad Software, La Jolla, CA, USA). Comparisons among multiple groups were conducted using one-way analysis of variance (ANOVA) followed by Tukey’s post hoc test. For pairwise comparisons, Student’s *t*-test was applied. A *p*-value < 0.05 was considered statistically significant.

For each experimental condition, the mean absorbance values obtained from the MTT assay and protein quantification were calculated, and the ratio of MTT/protein was determined. This normalization allowed for the evaluation of metabolic activity relative to total protein content, thereby distinguishing whether changes in MTT reduction reflected variations in cell number (biomass) or alterations in cellular metabolic activity.

## 4. Conclusions

This multidisciplinary investigation integrates ethnobotanical knowledge, phytochemical profiling, and biological evaluation to elucidate the therapeutic potential of *T. disciforme*. Ethnobotanical surveys conducted across three Iranian provinces highlight the relevance of this species in traditional medicine, particularly for gastrointestinal disorders. Untargeted metabolomic profiling revealed a chemically diverse extract composition, enriched in phenolic compounds and other bioactive secondary metabolites, with solvent polarity playing a decisive role in extraction efficiency and metabolite diversity. Supporting these traditional claims, antimicrobial assays demonstrated moderate effects of *T. disciforme* extracts, notably against *B. cereus*. While these findings substantiate the ethnomedical relevance of *T. disciforme* and advocate for its continued pharmacological exploration, we acknowledge certain limitations. The biological assessment addressed only a limited range of activities and therefore does not capture the full therapeutic potential of the species. This study focused on crude extracts, without further isolation of individual active compounds, which restricts the ability to attribute the observed effects to specific compounds. Studies focusing on cellular viability are of particular interest, as it seems that healthy and tumour cells behave differently when exposed to the same treatment. Nonetheless, collectively, our results provide a first step in bridging ethnobotanical knowledge and laboratory-based pharmacological evidence for *T. disciforme*. Future studies should include fractionation, bioassay-guided isolation, quantification of key compounds, and additional biological assays (e.g., anti-inflammatory, and safety assessments) to fully validate and expand the therapeutic potential of this medicinal plant.

## Figures and Tables

**Table 1:** Informant consensus factor (ICF).

Therapeutic Indications	Number of Used Reports (N_ur_)	Number of Taxa (N_t_)	ICF Value
Cardiac system	37	11	0.67
Urinary system	26	10	0.58
Nervous system	74	11	0.82
Respiratory disorder	9	3	0.63
Skin disorders	49	11	0.74
Digestive system	95	11	0.85
Wound healing	41	8	0.77

**Table 2:** Fidelity level (FL) values for the most cited medicinal plants.

Therapeutic Indications	Plant Species	FL (%)
Digestive system	*Tripleurospermum disciforme*	62%
	*Camellia sinensis*	76%
	*Echium amoenum*	25%
	*Mentha* spp.	100%
	*Cinnamomum zeylanicum*	85%
Skin disorders	*Tripleurospermum disciforme* *Thymus kotschyanus*	37% 30%
Wound healing	*Thymus kotschyanus*	50%
Nervous system	*Tripleurospermum disciforme* *Echium amoenum* *Stachys lavandulifolia*	38% 68% 77%
Urinary system	*Echium amoenum*	31%
Cardiac disorder	*Crocus haussknechtii*	73%
Respiratory disorder	*Zingiber officinale*	77%

FL = 100% (high fidelity level): Everyone who mentioned the plant used it for the same disease. This suggests strong reliability and specialized use. Lower FL values: The plant is used for multiple diseases, meaning it may not be strongly associated with just one condition.

**Table 3:** List of medicinal plants cited and their relative frequency citation (RFC) and use value (UV).

Plant Species	Number of Participants That Cited the Species	RFC	UV
*Tripleurospermum disciforme*	42	0.42	1.72
*Thymus kotschyanus*	40	0.4	1.75
*Echium amoenum*	32	0.32	1.31
*Mentha* spp.	30	0.3	1
*Crocus sativus* (Saffron)	15	0.15	1
*Camellia sinensis*	14	0.14	1
*Cinnamomum zeylanicum*	14	0.14	1
*Lavandula angustifolia*	10	0.1	1
*Stachys lavandulifolia*	9	0.09	1
*Malva sylvestris* L.	8	0.08	1
*Rosmarinus officinalis*	7	0.07	1
*Zingiber officinale*	7	0.07	1
*Ziziphora clinopodoides*	7	0.07	1
*Salvia* spp.	6	0.06	1
*Rosa* spp.	5	0.05	1
*Curcuma longa*	4	0.04	1
*Aloysia citrodora*	4	0.04	1
*Cichorium intybus*	2	0.02	1
*Eryngium planum*	2	0.02	1
*Salix aegyptiaca*	1	0.01	1
*Achillea* spp.	1	0.01	1
*Cuscuta epithymum*	1	0.01	1
*Ziziphus vulgaris*	1	0.01	1
*Citrus aurantium*	1	0.01	1
*Nigella arvensis*	1	0.01	1
*Elettaria cardamomum*	1	0.01	1
*Adiantum capillus-veneris* L.	1	0.01	1
*Foeniculum vulgare Mill.*	1	0.01	1
*Juniperus communis*	1	0.01	1
*Urtica urens*	1	0.01	1
*Cuminum cyminum*	1	0.01	1
*Syzygium aromaticum*	1	0.01	1
*Cassia angustifolia*	1	0.01	1
*Glycyrrhiza glabra* L.	1	0.01	1
*Portulaca oleracea* L.	1	0.01	1

**Table 4:** Compounds detected in *Tripleurospermum disciforme* (C.A.Mey.) Sch.Bip. extracts by UHPLC–timsTOF–MS analysis.

Compound Name	Formula	Measured *m*/*z*	Retention Time (min)	CCS (Å^2^)	Detected in These Solvents
2′,4′,6′-Trihydroxyacetophenone	C_8_H_8_O_4_	169.04974	6.54	120.8	Ethyl acetate, Hexane, Methanol
2-Acetylbenzoic acid	C_9_H_8_O_3_	163.04001	8.46	125.8	Ethyl acetate, Hexane, Methanol
3,4-Dicaffeoylquinic acid	C_25_H_24_O_12_	515.11879	9.57	211.5	Ethyl acetate
3-Feruloylquinic acid	C_17_H_20_O_9_	367.10357	8.85	188.7	Ethyl acetate, Hexane, Methanol
4-(3,4-Dimethoxyphenyl)-3-buten-1-ol	C_12_H_16_O_3_	209.11765	8.33	130.8	Ethyl acetate, Methanol
4-(4-Hydroxyphenyl)-2-butanone	C_10_H_12_O_2_	165.09078	11.66	121.0	Ethyl acetate, Hexane, Methanol
4-Hydroxycoumarin	C_9_H_6_O_3_	163.03905	6.2	116.2	Ethyl acetate, Hexane, Methanol
5-Hydroxymethyl-7-methoxybenzofuran	C_10_H_10_O_3_	179.07166	12.19	119.9	Ethyl acetate, Hexane, Methanol
6-Gingerol	C_17_H_26_O_4_	295.19226	14.38	155.9	Ethyl acetate, Hexane, Methanol
6-Hydroxyluteolin	C_15_H_10_O_7_	303.05216	9.26	153.2	Ethyl acetate, Hexane, Methanol
6-Hydroxyluteolin 7-*O*-rhamnoside	C_21_H_20_O_11_	447.09297	8.6	198.6	Ethyl acetate, Hexane, Methanol
7,3′,4′-Trihydroxyflavone	C_15_H_10_O_5_	269.04531	11.38	153.4	Ethyl acetate, Hexane, Methanol
9-Dehydroxyeurotinone	C_15_H_12_O_5_	271.06046	11.57	154.4	Ethyl acetate, Hexane, Methanol
Agrostophyllidin	C_17_H_16_O_4_	283.0973	13.03	171.6	Ethyl acetate, Hexane, Methanol
Altechromone A	C_11_H_10_O_3_	191.07067	10.22	123.7	Ethyl acetate, Hexane, Methanol
Caffeic acid	C_9_H_8_O_4_	179.03505	5.92	127.8	Ethyl acetate, Hexane, Methanol
Chrysin	C_15_H_10_O_4_	253.05016	12.84	152.2	Ethyl acetate, Hexane, Methanol
Deoxyarbutin	C_11_H_14_O_3_	195.10202	10.04	126.7	Ethyl acetate, Hexane, Methanol
Eriodictyol	C_15_H_12_O_6_	287.05448	10.72	161.3	Methanol
Kaempherol	C_15_H_10_O_6_	287.0567	11.17	148.6	Ethyl acetate, Methanol
Myricetin 3-*O*-rhamnoside	C_21_H_20_O_12_	463.08758	9.96	200.0	Ethyl acetate, Hexane, Methanol
N-Phenylacetylaminoacetic acid	C_10_H_11_NO_3_	194.08138	7.13	124.0	Ethyl acetate, Methanol
Neochlorogenic acid	C_16_H_18_O_9_	353.08764	0.83	180.7	Ethyl acetate, Hexane, Methanol
Olivetol	C_11_H_16_O_2_	181.1228	12.28	115.8	Hexane, Methanol
Scutellarein	C_15_H_10_O_6_	287.05658	10.79	128.5	Hexane, Methanol
p-HPEA-AC	C_10_H_12_O_3_	181.08631	4.25	108.7	Ethyl acetate

**Table 5:** Antioxidant properties of hexane, chloroform, ethyl acetate, and methanol extracts of *Tripleurospermum disciforme* (C.A.Mey.) Sch.Bip.

Samples	* DPPH Free Radical Scavenging Assay		
	IC_50_ (μg/mL)	** AAI	Antioxidant Activity
n-Hexane	290.020 ± 80.639	0.183 ± 0.013	No activity
Ethyl acetate	12.496 ± 4.153	4.211 ± 0.339	Very strong
Chloroform	71.984 ± 20.112	0.720 ± 0.050	Moderate
Methanol	64.774 ± 24.764	0.834 ± 0.068	Moderate
Gallic acid	3.923 ± 1.259	13.001 ± 0.672	Very strong

* DPPH—2,2-diphenyl-1-picrylhydrazyl; ** AAI—antioxidant activity index.

**Table 6:** Minimum inhibitory concentration (µg/mL) of hexane, chloroform, ethyl acetate, and methanol extracts of *Tripleurospermum disciforme* (C.A.Mey.) Sch.Bip.

	MIC (µg/mL)				
Strains	Hexane	Ethyl Acetate	Chloroform	Methanol	Tetracycline
*Staphylococcus aureus* ATCC 25923	1250	5000	5000	5000	2
*Escherichia coli* ATCC 25922	>5000	>5000	>5000	>5000	4
*Klebsiella pneumoniae* ATCC 13883	>5000	>5000	>5000	>5000	8
*Acinetobacter baumannii* LMG 1025	5000	5000	5000	>5000	2
*Bacillus cereus* ATCC 11778	2500	312	625	625	0.25
*Pseudomonas aeruginosa* ATCC 27853	>5000	>5000	>5000	>5000	16
*Salmonella Typhimurium* ATCC 13311	>5000	>5000	>5000	>5000	8
*Enterococcus faecalis* ATCC 29212	>5000	5000	>5000	>5000	16

## Data Availability

Data is contained within the manuscript and Supplementary Material.

## Supplementary Materials

The following supporting information can be downloaded at https://www.mdpi.com/article/10.3390/molecules30183685/s1: Table S1. Annotated phytochemical compounds identified in the extracts of *Tripleurospermum disciforme* using untargeted UHPLC–timsTOF–MS analysis. Compound annotations were performed based on spectral library matching (SL), an in-house analyte list (AL) of phenolic compounds, and SmartFormula (SF) calculations. The table includes the compound name, molecular formula, accurate m/z, retention time (RT), collision cross section (CCS), and the solvents in which each compound was detected (methanol, ethyl acetate, and hexane); Table S2: Diameter of inhibition zone (mm) of methanol, hexane, ethyl acetate, and chloroform extracts of *Tripleurospermum disciforme*.
